# DOING AGE AS MARGINALIZATION AND EXCLUSION: ON AGE CODING AND THE CONSTRUCTION OF AGE NORMALITIES AND AGEISM

**DOI:** 10.1093/geroni/igz038.2108

**Published:** 2019-11-08

**Authors:** Clary Krekula

**Affiliations:** Karlstad University, Karlstad, Sweden

## Abstract

Early definitions of ageism as prejudice, attitudes and discrimination tend to stress the cognitive aspects of the processes where groups of people are being marginalized and/or excluded because of their age. This differ from recurrent studies which emphazise social categorization such as age and gender as a dynamic social positioning practice and as a complex form of doing age as marginalization and exclusion. Based on qualitative interviews with eleven men between the ages of 56 and 74, who work with manual labour at a steel company in Sweden, this paper discusses the processes where age normality and ageism are constructed in parallel. Departing from the concept age coding; distinctive practices associating a context or a phenomenon with demarcated ages, it shows the disciplining dimension of ageism. The paper concludes with a reflection on the central position which ageism can have in neo-liberal governance by creating social insecurity among older workers.

